# Oral Susceptibility of Singapore *Aedes* (*Stegomyia*) *aegypti* (Linnaeus) to Zika Virus

**DOI:** 10.1371/journal.pntd.0001792

**Published:** 2012-08-28

**Authors:** MeiZhi Irene Li, Pei Sze Jeslyn Wong, Lee Ching Ng, Cheong Huat Tan

**Affiliations:** Environmental Health Institute, National Environment Agency, Singapore, Singapore; USAMRIID, United States of America

## Abstract

**Background:**

Zika virus (ZIKV) is a little known flavivirus that caused a major outbreak in 2007, in the South-western Pacific Island of Yap. It causes dengue-like syndromes but with milder symptoms. In Africa, where it was first isolated, ZIKV is mainly transmitted by sylvatic *Aedes* mosquitoes. The virus has also been isolated from *Ae. aegypti* and it is considered to be the vector involved in the urban transmission of the virus. Transmission of the virus by an African strain of *Ae. aegypti* has also been demonstrated under laboratory conditions. The aim of the present study is to describe the oral susceptibility of a Singapore strain of *Ae. aegypti* to ZIKV, under conditions that simulate local climate.

**Methodology/Principal Findings:**

To assess the receptivity of Singapore's *Ae. aegypti* to the virus, we orally exposed a local mosquito strain to a Ugandan strain of ZIKV. Upon exposure, fully engorged mosquitoes were maintained in an environmental chamber set at 29°C and 70–75% RH. Eight mosquitoes were then sampled daily from day 1 to day 7, and subsequently on days 10 and 14 post exposure (pe). The virus titer of the midgut and salivary glands of each mosquito were determined using a tissue culture infectious dose_50_ (TCID_50_) assay. High midgut infection and salivary gland dissemination rates were observed. By day 5 after the infectious blood meal, ZIKV was found in the salivary glands of more than half of the mosquitoes tested (62%); and by day 10, all mosquitoes were potentially infective.

**Conclusions/Significance:**

This study showed that Singapore's urban *Ae. aegypti* are susceptible and are potentially capable of transmitting ZIKV. The virus could be established in Singapore should it be introduced. Nevertheless, Singapore's current dengue control strategy is applicable to control ZIKV.

## Author Summary

Zika virus (ZIKV) is an emerging mosquito-borne zoonotic pathogen that causes dengue-like syndromes. Despite its high epidemic potential, little is known about the virus. Although the isolation of the virus was confined to the African continent, serological evidences have shown the widespread distribution of ZIKV, particularly in Asia. In 2007, it caused a major outbreak on the Pacific Island of Yap, infecting more than 70% of the island’s inhabitants. The propensity of the virus to spread outside its known geographical range was again demonstrated when it was detected in the US from travellers coming back from endemic countries. Several species of *Aedes* spp. mosquitoes have been incriminated as vectors of ZIKV, including *Ae. aegypti*. The current study showed that local *Ae. aegypti* are highly susceptible to ZIKV and by day 5 post-infectious blood meal, more than 50% of mosquitoes were potentially infective. Singapore being a tourist and a business hub, coupled with the presence of susceptible vector and a population that is immunologically naive and vulnerable, the local transmission of the ZIKV is plausible. Nevertheless, Singapore's current dengue control strategy is applicable to control ZIKV.

## Introduction

Zika virus (ZIKV) is an emerging mosquito-borne pathogen belonging to the genus *Flavivirus* of the Family *Flaviviridae* [1]. It is a positive single stranded RNA virus with a 10,794 nucleotide genome that is closely related to the Spondweni virus (*Flavivirus*, Family *Flaviviridae*) [2], [3]. The virus was first isolated in 1947 from a febrile rhesus monkey in the Zika forest of Uganda [4]. Non-human primates were implicated as the reservoir host of ZIKV in Africa and Asia [5].

In humans, ZIKV causes a mild infection manifested by a rash, fever, joint and muscle pain, headache and peri-orbital pain, which are characteristic signs and symptoms of flavivirus infections [6], [7]. The first human ZIKV infection was reported in Uganda in 1964 [6]. Although the isolation of ZIKV has so far been confined to the African continent [8], [9], serological evidence has shown widespread distribution of the virus even in Asian countries such as Malaysia, India, Philippines, Thailand, Vietnam, Indonesia, and Pakistan [10], [11], [12], [13], [14], [15]. The first major outbreak of human ZIKV infection was reported in the Pacific island of Yap and its adjoining islands in the Federated State of Micronesia in 2007 [3], [7], [16], [17]. The outbreak lasted four months infecting approximately 73% of the islands' population [7]. In 2011, ZIKV was first reported in the western hemisphere in travellers returning from Senegal [18]. Most recently, ZIKV was isolated from a 3-year old boy in Cambodia in 2010 [19].

ZIKV is transmitted to humans by *Aedes* spp. mosquitoes. The earliest evidence of ZIKV in a pool of *Ae. africanus* from Uganda in 1948 coincides with its first isolation from a rhesus monkey in the same location [4]. Subsequent documents reported further isolation of the virus from *Ae. africanus* and *Ae. apicoargenteus* caught in the Zika forest [20], [21], [22]; from *Ae. luteocephalus* in Nigeria in 1969 [23]; and from *Ae. vitattus*, *Ae. furcifer*, and *Ae. aegypti* in Ivory Coast in 1999 [24]. High prevalence of ZIKV antibodies in the urban population of Nigeria has led Fagbami [23] to suspect that *Ae. aegypti* may play an important role in the urban transmission of ZIKV. Further evidence came from Asia, when ZIKV was isolated from a pool of *Ae. aegypti* caught in Bentong, Peninsular Malaysia [25]. This finding provided evidence of ZIKV transmission outside Africa. In Indonesia, the peak of human ZIKV infections coincides with peak *Ae. aegypti* population which is by the end of rainy season [14]. Apart from field surveillance data, early experimental studies conducted by Boorman and Porterfield [26] and Cornet et al. [27] have also demonstrated the competency of *Ae. aegypti* to transmit ZIKV.

Considering the geographic spread and the possible impact on susceptible human populations, mosquito-borne diseases are currently considered as a major threat to global health in both developing and developed world [28], [29]. According to Gushulak et al. [30], the threat of emerging infectious diseases is mainly influenced by the migration and mobility of the human populations. The dengue, chikungunya and malaria situations in Singapore clearly demonstrate the role of importation in shaping the epidemiology of these diseases [31], [32], [33]. Introduction of ZIKV into Singapore, a travel and trading hub, is plausible. Coupled with the local presence of *Ae. aegypti*, local transmission of the virus is likely. Furthermore, as ZIKV has never been reported in Singapore, the local population is presumed to be immunologically naive and vulnerable to the infection.

Although experimental studies conducted in the past have shown that *Ae. aegypti* is a competent vector for ZIKV, these studies used African strains of *Ae. aegypti* that were caught in Nigeria [26] and Senegal [27] and had been maintained in the laboratory for years. Furthermore, experimental methods used in these studies differed from those of the current study. Although Boorman and Porterfield [26] infected the mosquitoes using the oral route, the average incubation temperature was 24°C, which is low in the tropical context and resulted in an extrinsic incubation period that suggested low vectorial capacity. While Cornett et al. [27] incubated their infected mosquitoes between 27 to 28°C, the method of infection was by intrathoracic route which can artificially lead to shorter extrinsic incubation period and higher number of mosquitoes infected. In addition, the geographical variations in terms of oral susceptibility of mosquitoes to different viruses are also well documented [34], [35], [36], [37], [38], [39]. The present study describes the oral susceptibility of a Singapore field strain *Ae. aegypti* to ZIKV, under condition that simulate local climate.

## Materials and Methods

### Mosquitoes

*Ae. aegypti*, used for the experimental infection, were derived from eggs collected in the Western part of Singapore during a weekly ovitrap surveillance study to determine mosquito population density. Ovitraps were placed in public areas, mostly along the common corridors of public housing. The surveillance study was conducted by colleagues from the Environmental Health Institute. F_0_ adults were allowed to emerged and were maintained under standard insectary condition at 28±1°C and 75–80% relative humidity (RH), with a photoperiod of 12h∶12h light∶dark (L∶D) cycles. They were allowed to mate randomly and fed with pathogen-free pig's blood (A*star Biomedical Resource Center, Singapore) using a Hemotek membrane feeding system (Discovery Workshops, Lancashire, United Kingdom). F_1_ eggs were collected using filter paper (Whattman, USA). Eggs were then allowed to hatch using de-chlorinated water and larvae were reared in 25 cm×30 cm×9 cm enamel pans containing 800 mL of water and fed with crushed dog food. Pupae were placed in 30 cm×30 cm×30 cm (HxWxL) cages before emergence. Prior to the infectious feed, adult mosquitoes were provided with 10% sugar/Vitamin B complex solution *ad libitum*.

### Virus

Ugandan MR766 ZIKV strain obtained from the American Type Culture Collection (Manassas, VA, USA) was used to expose the mosquitoes to ZIKV. This virus was originally isolated from the blood of an experimental sentinel rhesus monkey in 1947 [4] and passaged in suckling mouse brains. The stock virus used in the current study has been passaged thrice in Vero cells prior to the infectious feed.

### Oral infection of mosquitoes

Five- to 7-day-old female mosquitoes (n = 120) were transferred to 0.5 L containers and starved for 24 hours prior to the infectious blood meal. The blood meal consisted of 1∶1 100% swine-packed RBC (Innovative Research, USA) and fresh virus suspension at a final concentration of 7.0 Log_10_ tissue culture infectious dose_50_ (TCID_50_)/mL. Adenosine Triphosphate (Fermentas, USA), at a final concentration of 3 mM, was added to the blood meal as a phagostimulant. Mosquitoes were fed with an infectious blood meal that was constantly warmed to 37°C using a Hemotek membrane feeding system (Discovery Workshops) housed in a feeding chamber. Thirty minutes after exposure to the infectious blood meal, mosquitoes were cold anesthetized at −20°C. Fully engorged females were transferred to 300 mL cartons and were maintained in an environmental chamber (Sanyo, Japan) at 29°C and 70–75% RH with a 12h/12h L∶D cycle and provided with 10% sugar/vitamin B complex *ad libitum*. All experiments were carried out in an arthropod containment level 2 (ACL-2) facility.

### Mosquito processing

To determine the ZIKV infection and dissemination rates in *Ae. aegypti*, eight mosquitoes were sampled daily from day 1 to day 7, and subsequently on days 10 and 14 post exposure (pe). To prevent cross-contamination of virus between midgut and salivary glands of each mosquito, these organs were carefully dissected using different dissecting needles and the organs were rinsed in Medium 199 (M199) (Gibco, USA) supplemented with amphotericin B (Sigma Aldrich, USA). The midguts and salivary glands from each mosquito were individually transferred to 2 mL microtubes containing 250 µL of M199. These organs were then homogenized using five mm stainless steel grinding balls (Retsch, Germany) in a MM301 mixer mill (Retsch, Germany) set at frequency of 12/sec for 1 min. The supernatant of the homogenate was applied in the viral titer assay. All dissecting needles were dipped in 80% ethanol and cleaned before being re-used. All experiments were conducted inside an ACL-2 facility.

### Tissue Culture Infectious Dose_50_ Assay

Viral titers in this study were determined with a tissue culture infectious dose_50_ assay, an endpoint dilution technique, using Vero cells as described by Higgs et al. [40]. Briefly, 100 µL of 10-fold serial dilutions of each sample were titrated (in duplicate) in 96-well microtititer plates and incubated with Vero cells at 37°C and 5% CO_2_. At the end of day-7 incubation, the cells were examined microscopically for ZIKV-induced cytopathic effect (CPE). A well is scored positive if any CPE is observed compared to the uninfected control cells. All virus titers were expressed as Log_10_ TCID_50_/mL.

### Statistical analysis

Proportion infected was calculated by dividing the number of infected midguts (or salivary glands) by the total number of miguts (or salivary glands) sampled. To compare viral titers at different time points, raw data was subjected to a normality test using SPSS Ver 18 (IBM, USA). Data that passed the normality test were analyzed by analysis of variance using the above mentioned software.

## Results

### Oral susceptibility of *Ae. aegypti* to ZIKV

**Figure 1:**
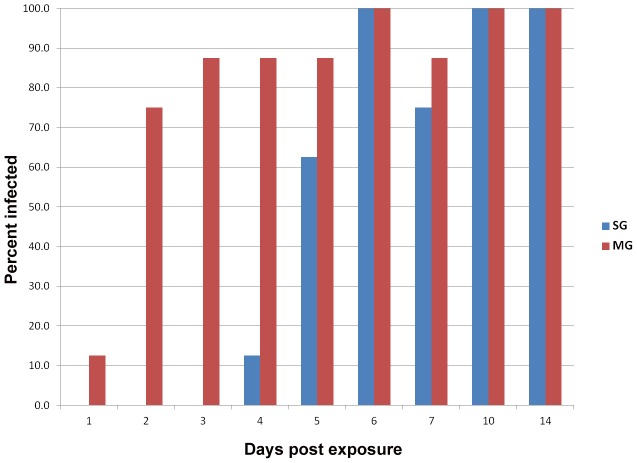
Midguts and salivary glands infection rates of ZIKV in *Ae. aegypti* at different days post-infectious bloodmeal. Eight mosquitoes were sampled per day.

Presence or absence of blood in the midgut was verified during dissection under a Stereoscope (Olympus, USA). By Day 3, when blood had been completely digested, seven (87.5%) of the analyzed mosquitoes were positive for ZIKV (Figure 1). From day 6 pe onwards, all midguts were positive for ZIKV except for one of the mosquitoes that was negative for the virus at day 7-pe.

The presence of viable ZIKV in the salivary glands (n = 1) was first observed on day 4 pe (Figure 1) and 62% of mosquitoes sampled on day 5-pe showed detectable virus in the salivary glands. ZIKV was observed in salivary glands of all infected mosquitoes sampled at days 10 and 14 pe.

### ZIKV midgut and salivary gland titers

**Figure 2:**
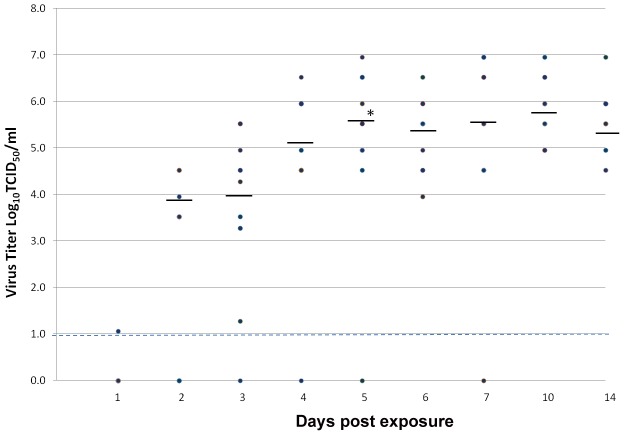
Titer of ZIKV in midguts of *Ae. aegypti* at different days post-infectious bloodmeal. The bar indicates median viral titers and limit of detection is represented by broken lines. A significant increase(*) (P = 0.026) in mean viral titer was observed between days 3 and 5 pe.

Figure 2 presents ZIKV midgut titers at different days pe. Although remaining blood meal in midgut was not removed, an eclipse phase typically associated with low virus midgut titer can be seen on day 1 pe, with only one of the midgut showing detectable ZIKV. Virus titers in day 2 pe were higher than that observed for day 1 pe, mirroring the results obtained on percentage of midguts infected (Figure 1). These suggest that midgut ZIKV titer observed during day 2 pe was most probably due to virus replication in the midgut rather than to the remaining amount of blood observed in some of the mosquitoes. A significant increase (P<0.026) in mean viral titers was observed between days 3 pe (3.9 Log_10_ TCID_50_/mL) and day 5 pe (5.6 Log_10_ TCID_50_/mL). From day 6 pe onwards, mean viral titers showed a decreasing trend from fluctuated between 5.4 Log_10_ TCID_50_/mL and 5.9 Log_10_ TCID_50_/mL but the differences observed were not statistically significant (P≥0.91).

**Figure 3:**
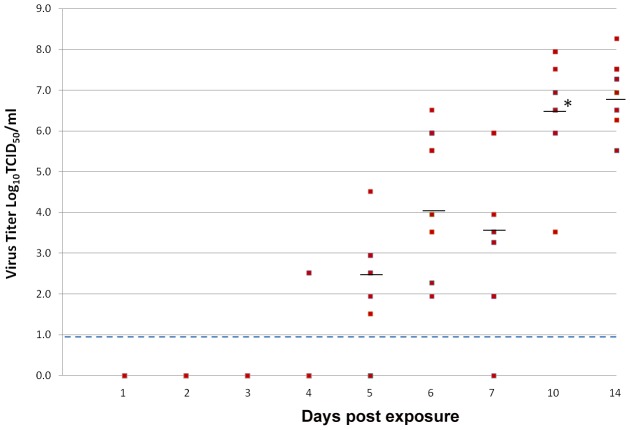
Titer of ZIKV in the salivary glands of *Ae. aegypti* at different days post-infectious bloodmeal. The bar indicates median viral titers and limit of detection is represented by broken lines. A significant increase(*) (P<0.001) in mean viral titer was observed between days 7 and 10 pe.

ZIKV titers in the salivary glands increased from day 4 pe onwards (Figure 3). Although the difference in mean viral titers from day 5 pe (2.7 Log_10_ TCID_50_/mL) to day 7 pe (3.7 Log_10_ TCID_50_/mL) was not significant (P = 0.68), the mean viral load increased significantly (P<0.001) by day 10 pe (6.4 Log_10_ TCID_50_/mL), achieving the highest mean viral load of >8.0 Log_10_ TCID_50_/mL by day 14 pe.

## Discussion

Recent unprecedented spread of chikungunya virus (CHIKV) in many parts of the world, with millions of people affected, exemplifies how arboviruses can adapt and affect human health on a global scale [31]. Singapore's vulnerability to emerging and re-emerging arboviruses is accentuated by the country's location as a popular tourist and business hub, high dependency on migrant workers, tropical climate, dense population, and the presence of potential mosquito vectors. An outbreak of chikungunya in Singapore during the 2008–09 period attests the country's vulnerability to mosquito-borne diseases [31], [41]. The outbreaks of ZIKV on Yap Island and the worldwide spread of CHIKV have shown the propensity of arboviruses to spread outside their known geographical range and their potential to cause large-scale epidemics.

Unlike CHIKV which has received much scientific attention, ZIKV is a little-known flavivirus despite its outbreak potential [42]. Most studies on ZIKV were conducted more than two decades ago and there is a dearth of information on mosquito-ZIKV interactions that are salient to a better understand virus transmission. In 1956, Boorman and Porterfield [26] successfully transmitted the virus to both mice and monkeys using ZIKV-infected laboratory strains of *Ae. aegypti*. Cornet et al. [27] further demonstrated that a high percentage (88%) of intrathoracically infected *Ae. aegypti* can transmit ZIKV to experimental mice within 7 days and transmission rates increased up to 95% on day 21 pe. The current study, using a field strain of mosquitoes, showed that Singapore's *Ae. aegypti* are highly susceptible to ZIKV, with high midgut infection and salivary gland dissemination rates. By day 5 pe, 62% of the mosquitoes had detectable ZIKV in their salivary glands and by day 10 pe all mosquitoes were potentially infective. Based on the studies of Cornet et al. [27], nearly all mosquitoes with ZIKV in their salivary glands are assumed to be able to transmit the virus. This is supported by previous studies that have shown oral transmission of dengue (DENV) [43], [44] and West Nile (WNV) [45] viruses were correlated with the proportion of mosquitoes with infected salivary glands.

The decrease in midgut viral titer at day 14 pe observed in our study was consistent with other published DENV and WNV studies [46], [47], [48] and were probably due to virus clearance by the mosquito immune system [47], [49], [50]. Despite a decrease in midgut viral titer, ZIKV infection in salivary glands was found to be higher than that observed in midgut. This suggests that the proliferation of ZIKV in *Ae. aegypti* salivary glands is not attributed to direct dissemination from the midgut, but rather a result of viral dissemination and amplification within the glands or other organs or tissues such as hemocytes, ganglion, fat bodies etc [49], [51], [52]. Salivary gland dissemination rates obtained from our current study is similar to that observed for a local highly epidemic DENV-2 in the same strain of *Ae. aegypti* (Tan et al., unpublished data).

A phylogenetic analysis, based on the NS5 region, of ZIKV revealed three branches: West African (Nigeria), East African (Uganda) and those from Yap island (ZIKV 2007 EC), with the latter virus being the most distally related [17]. The strain used in our current study, MR766, is the Ugandan prototype strain and the only strain available to our laboratory. It would be very interesting to study and compare the recent epidemic ZIKV 2007 EC strain in *Ae. aegypti*, especially in the light of a four amino acid motif found in the viral envelope genes of the ZIKV 2007 EC strain that are absent in the MR766 strain [17]. Unfortunately, no ZIKV 2007 EC was isolated during the outbreak in Yap Island. The four amino acid motif found in the ZIKV 2007 EC strain correspond to an envelope protein 154 glycosylation motif and the loss of this motif in the ZIKV prototype strain is thought to have been due to extensive passage in mice [17]. Studies have showed that loss of glycosylation motif due to mutation has been found to affect the replication rates of tick-borne encephalitis virus. DENV, and WNV in both vector hosts and insect cell lines and the dissemination rate of WNV in different *Culex* spp. mosquitoes [53], [54], [55], [56]. Despite the absence of this aa 154 glycan, the present study has shown that ZIKV MR766 has a high dissemination rate in Singapore's *Ae. aegypti*. This could probably be due to the high midgut pH found in *Ae. aegypti* [57], a characteristic shared by *Cx. tarsalis*, which rendered it susceptible to WNV virus lacking the aa 154 glycan [53]. Future studies with other strains will take these observations into consideration.

Timely detection of the causative agents and implementation of effective control strategies during an epidemic or outbreak are always challenging. A fully-integrated vector control program incorporating advances in laboratory techniques and surveillance programs designed to address all components of the virus life cycle is considered the best approach in detecting and controlling any vector-borne disease as they emerge [42]. Such was the case of the successful control of the CHIKV outbreak in Singapore in 2008 [41]. The use of rapid and sensitive diagnostic and effective field surveillance tools and good coordination between field and laboratory personnel coupled with an understanding of mosquito-virus relationship assisted in the situation assessment and operational decision-making in controlling the outbreak.

The present study revealed the potential role of local *Ae. aegypti* as a vector of ZIKV. Given the presence of the virus in the region, the Environmental Health Institute screened febrile cases not attributable to DENV and CHIKV for ZIKV and other arboviruses. Among the 690 cases screened between 2009 and 2010, none was found positive for flaviviruses other than DENV. While there is currently no evidence of its circulation in Singapore, regular screening will be performed to monitor the situation. Based on the information gathered from this study (e.g. viral dissemination rate), the threat of ZIKV can be addressed by the existing dengue control programme. However, there is also a need to determine the susceptibility of other common mosquito species, in order to design a comprehensive vector control strategy for Zika infection.
